# Nomogram for predicting the probability of rectal anastomotic re-leakage after stoma closure: a retrospective study

**DOI:** 10.1186/s12885-024-12544-8

**Published:** 2024-07-12

**Authors:** Yuegang Li, Gang Hu, Jinzhu Zhang, Wenlong Qiu, Shiwen Mei, Xishan Wang, Jianqiang Tang

**Affiliations:** Department of Colorectal Surgery, National Cancer Center/National Clinical Research Center for Cancer/Cancer Hospital, Chinese Academy of Medical Sciences and Peking Union Medical College, Beijing, 100021 China

**Keywords:** Rectal cancer surgery, Anastomotic re-leakage, Stoma closure, Nomogram

## Abstract

**Background:**

In this study, we aimed to identify the risk factors in patients with rectal anastomotic re-leakage and develop a prediction model to predict the probability of rectal anastomotic re-leakage after stoma closure.

**Methods:**

This study was a single-center retrospective analysis of patients with rectal cancer who underwent surgery between January 2010 and December 2020. Among 3225 patients who underwent Total or Partial Mesorectal Excision (TME/PME) surgery for rectal cancer, 129 who experienced anastomotic leakage following stoma closure were enrolled. Risk factors for rectal anastomotic re-leakage were analyzed, and a prediction model was established for rectal anastomotic re-leakage.

**Results:**

Anastomotic re-leakage after stoma closure developed in 13.2% (17/129) of patients. Multivariable analysis revealed that neoadjuvant chemoradiotherapy (odds ratio, 4.07; 95% confidence interval, 1.17–14.21; *p =* 0.03), blood loss > 50 ml (odds ratio, 4.52; 95% confidence interval, 1.31–15.63; *p* = 0.02), and intersphincteric resection (intersphincteric resection vs. low anterior resection: odds ratio, 6.85; 95% confidence interval, 2.01–23.36; *p* = 0.002) were independent risk factors for anastomotic re-leakage. A nomogram was constructed to predict the probability of anastomotic re-leakage, with an area under the receiver operating characteristic curve of 0.828 in the cohort. Predictive results correlated with the actual results according to the calibration curve.

**Conclusions:**

Neoadjuvant chemoradiotherapy, blood loss > 50 ml, and intersphincteric resection are independent risk factors for anastomotic re-leakage following stoma closure. The nomogram can help surgeons identify patients at a higher risk of rectal anastomotic re-leakage.

## Background

With continuous advances in minimally invasive surgery and comprehensive treatments, an increasing number of patients with ultralow rectal cancers can maintain bowel continuity [1, 2]. However, this trend has increased the risk of anastomotic leakage (AL) and increasing numbers of surgeons are utilizing diverting stoma (DS) to mitigate the severe repercussions of AL [3–5]. Nevertheless, the incidence of AL after rectal surgery still ranges from 3 to 15% [6, 7] and can exceed 20% after neoadjuvant chemoradiotherapy (nCRT) and intersphincteric resection (ISR) surgery [3, 8]. Even with the closure of a DS, the risk of AL recurrence occurs, a condition referred to as “anastomotic re-leakage.”

The indication to close a DS is typically based on the absence of leak detection on imaging and colonoscopy, the absence of symptoms, and considering the patient’s recovery and willingness. Current research suggests that stoma closure is performed between 3 and 6 months after surgery, although some studies propose an earlier closure [9–11]. There remains a debate about the indications for stoma closure in patients experiencing AL. Hain suggests that asymptomatic patients with AL should undergo stoma closure 6 months after the initial surgery, but 16% of patients experienced anastomotic re-leakage [12]. Kitaguchi’s [13] study analyzed factors associated with ISR surgery contributing to anastomotic re-leakage following stoma closure. However, the sample size was limited, with only 69 cases experiencing Clavien-Dindo Grade III or higher rectal anastomotic leakage after stoma closure.

The aim of this study, comprising patients with maximal re-leakage, was to assess the risk factors for rectal anastomotic re-leakage and develop a prediction model for the probability of anastomotic re-leakage following stoma closure.

## Methods

### Patients

Data from our database and medical records of patients treated at Cancer Hospital, Chinese Academy of Medical Sciences, were reviewed from January 2010–December 2020. The patient inclusion criteria were as follows: (1) preoperative pathological examination confirming rectal adenocarcinoma; (2) underwent laparoscopic surgery, including anterior resection (AR), low anterior resection (LAR), or ISR surgery; and (3) confirmed AL during hospitalization or recovery period. The exclusion criteria were: (1) emergency surgery for acute intestinal obstruction, bleeding, or perforation; (2) subtotal colectomy or total colectomy due to multiple primary tumors; and (3) individuals with distant organ metastasis. All patients provided written informed consent, and the study was approved by the Ethics Committee of Cancer Hospital, Chinese Academy of Medical Sciences (ethical approval number 22/503–3705).

### Treatment procedures

For patients with preoperative stage cT3/N + advanced low rectal cancer, nCRT is recommended as a routine treatment. All operations were performed by experienced surgical teams, regardless of whether the anastomosis was stapled or hand-sewn, and a diverting ileostomy was routinely considered in ISR and nCRT patients. After the index surgery, basic judgments are made based on the patient’s vital signs, laboratory results, and whether there are any abnormal signs in the abdomen or drainage shape. Once AL is suspected, computed tomography (CT) or endoscopy is performed for further diagnosis. Protective measures such as active anti-infection, maintaining unobstructed drainage, and systemic nutritional support therapy can be considered for patients with ileostomy. For patients without ileostomy, secondary surgery is performed on the transverse colon or ileum stoma if there is apparent peritonitis or shock. The healing of the anastomosis in all patients with AL is evaluated every month in our outpatient clinic, with the first choice being an internal anal examination. If necessary, iodine oil imaging and CT scans are also used for further evaluation. Once the anastomosis is found to be completely healed and meets the healing standards, a stoma closure operation is usually performed. Following stoma closure, all patients receive regular follow-up every other month. Colonoscopy and CT examinations are conducted every 30–90 days, and magnetic resonance imaging is performed if necessary. For patients who experience re-leakage, anti-infection treatment is proactively administered. In cases where patients do not respond to conservative treatment, re-stoma surgery may be considered.

### Diagnostic criteria for AL, clinical healing of AL, and re-leakage following stoma closure

All ALs were confirmed radiologically or endoscopically and assessed according to the Clavien–Dindo classification [14]. We included patients experiencing AL with stoma closure. The three criteria for clinical healing of AL comprised the following: (1) water-soluble contrast imaging shows no anastomotic stenosis, contrast extravasation, diverticula, or sinus formation; (2) colonoscopy confirms the integrity of anastomosis without any defects; and (3) CT scan confirms the absence of gas or fluid accumulation around the anastomotic site. Diagnostic criteria for anastomotic re-leakage after rectal anastomosis consisted of any of the following conditions being present: (1) digital rectal examination or colonoscopy reveals an incomplete anastomotic ring, anastomotic dehiscence, fistula, or sinus formation; (2) imaging studies such as CT or magnetic resonance imaging (MRI) confirm the discontinuity of the colonic wall at the anastomotic site or presence of fluid collection, abscess, and gas shadow around the anastomosis; (3) imaging with water-soluble contrast agent shows contrast extravasation from the anastomotic site into the extraluminal space; (4) persistent perianal abscess or anal fistula; and (5) negative imaging findings but the presence of vaginal or urethral gas or fecal discharge symptoms.

### Data collection and analysis

The clinical and pathological characteristics of patients were collected. IBM SPSS Statistics version 26 (IBM Corp., Armonk, NY, USA) and R software version 4.0 (R Foundation for Statistical Computing, Vienna, Austria) were used for statistical analyses. Pearson’s chi-square and Fisher’s exact tests were used to compare categorical variables. The odds ratio (OR) and 95% confidence interval (CI) of risk factors were analyzed using logistic regression. All statistical analyses were two-sided, and statistical significance was set at *p* < 0.05. The results of the multivariable analysis in the cohort are presented in a nomogram. For internal validation, 1000 bootstrap resamples were used to calculate the Harrell consistency index (c-index) [15]. We assessed the predictive power of the nomogram using receiver operating characteristic (ROC) curve analysis. A calibration curve was used to explore the performance of the nomogram.

## Results

### Patient characteristics

**Fig. 1:**
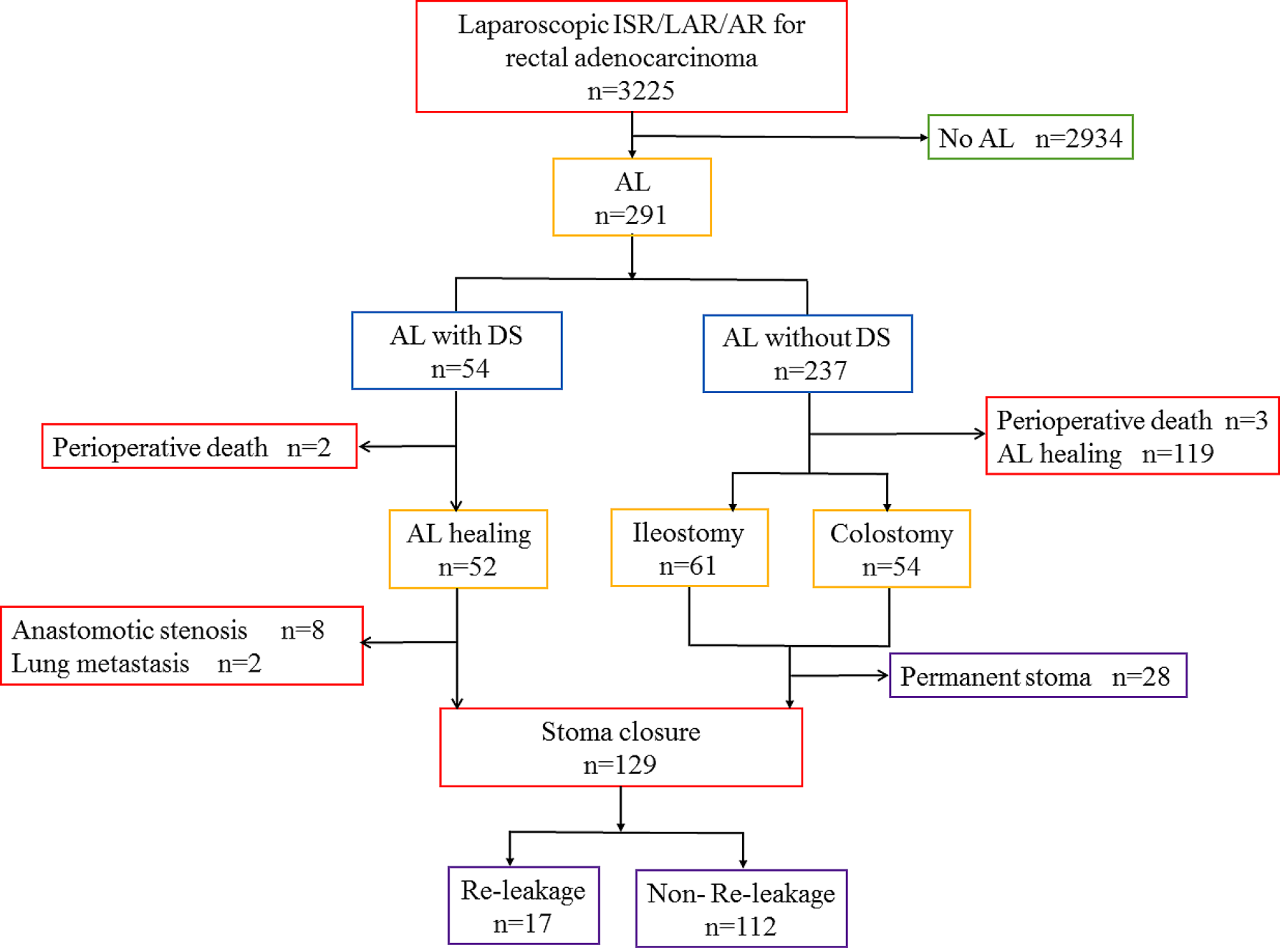
Flowchart of patient selection

Among the 3,225 patients who underwent rectal cancer surgery, 291 (9.0%) experienced AL: 54 patients (18.6%) with AL who had a DS and 237 (81.4%) with no DS. Forty-two patients were treated through non-operative treatment and finally received secondary surgery to close the stoma in patients with AL with DS. Among 237 patients without a protective stoma, 119 patients experiencing a minor leakage were healed through conservative treatment without ostomy surgery, and 87 patients received secondary surgery to close the stoma in patients with AL without DS. Ultimately, 129 patients who met the criteria for AL healing underwent secondary surgery to close the stoma. The study flowchart is illustrated in Fig. 1, and Table 1 provides a summary of baseline characteristics of the patients.

**Table 1:** Basic characteristics and univariate analysis of re-leakage in patients with stoma closure

Characteristics	Un- Re-leakage *n* = 112	Re-leakage *n* = 17	Univariate analysis	
			OR (95% CI)	*p* value
Sex, male	88 (78.6)	11 (64.7)	2.00 (0.67–5.96)	0.21
Age > 60 years	35 (31.3)	7 (41.2)	1.54 (0.54–4.38)	0.42
BMI > 25 kg/m^2^	68 (60.7)	11 (64.7)	1.19 (0.41–3.44)	0.75
Drug history (Yes)	13(11.6)	4(23.5)	2.34(0.66–8.27)	0.19
Smoke (Yes)	43(38.4)	6(35.3)	0.88(0.30–2.54)	0.81
Diabetes	16 (14.3)	2 (11.8)	0.80 (0.17–3.84)	0.78
ASA				
I–II	103 (92.0)	13 (76.5)	0.28 (0.78–1.05)	0.06
III	9 (8.0)	4 (23.5)		
nCRT	19 (17.0)	8 (47.1)	4.35 (1.49–12.72)	0.007
Blood loss>50 ml	27 (24.1)	9 (52.9)	3.54 (1.24–10.08)	0.02
LCA preservation	13 (11.6)	4 (23.5)	2.34 (0.66–8.27)	0.19
Type of surgery				0.004
LAR	75 (67.0)	6 (35.3)	Reference	
ISR	21 (18.8)	11 (64.7)	6.55 (2.17–19.79)	0.001
AR	16 (14.3)	0 (0)	-	1.00
Tumor size>35 mm	79 (61.2)	9 (52.9)	0.68 (0.24–1.88)	0.45
Differentiation				
Poor	31 (27.7)	2 (11.8)	0.35 (0.08–1.61)	0.16
Well/Moderate	81 (72.3)	15 (88.2)		
(y) pTNM stage				
I–II	61 (54.5)	10 (58.8)	0.84 (0.30–2.36)	0.74
III	51 (45.5)	7 (41.2)		
ALB < 35 g/L	6 (5.4)	2 (11.8)	2.36 (0.44–12.76)	0.32
CEA ≥ 5 ng/ml,	28 (25.0)	5 (29.4)	1.25 (0.41–3.86)	0.70
Timing of testing anastomotic integrity, months, IQR	3.5(2.9-4.0)	3.7(3.3–4.2)	-	0.72
Timing of stoma closure, months, IQR	10 (8–12)	9 (6–13)	-	0.56
Clavien–Dindo grade of initial AL			1.67(0.51–5.41)	0.40
I/II	38(33.9)	4(23.5)		
III/IV	74(66.1)	13(76.5)		
Chronic presacral abscess of initial AL	12(10.7)	4(23.5)	2.56(0.72–9.14)	0.15

*Abbreviations*: BMI, body mass index; ASA, American Society of Anesthesiologists; nCRT, neoadjuvant chemotherapy; AR, anterior resection; LAR, low anterior resection; ISR, intersphincteric resection; (y) pT4/N+, pathologic T4 stage or having positive lymph nodes retrieved with or without neoadjuvant therapy; CEA, Carcinoembryonic antigen; CI, confidence interval; OR, odds ratio; IQR, interquartile range; Drug history, steroids or immunosuppressive treatment

### Risk factors for anastomotic re-leakage after stoma closure

A total of 129 patients with AL underwent stoma closure between 3 and 17 months after meeting the clinical healing criteria. Among them, 17 patients (13.2%) experienced rectal anastomotic re-leakage within 1–11 months after the stoma closure. Univariate analysis revealed that several factors influenced anastomotic re-leakage after closure, including ASA score (OR = 0.28, 95% CI: 0.78–1.05, *p =* 0.06), blood loss exceeding 50 ml (OR = 3.54, 95% CI: 1.24–10.08, *p =* 0.02), nCRT (OR = 4.35, 95% CI: 1.49–12.72, *p =* 0.007), and type of surgery (ISR vs. LAR: OR = 6.55, 95% CI: 2.17–19.79, *p =* 0.001). In contrast, sex, BMI, diabetes status, preservation of LCA, tumor size, differentiation, time-to-stoma-closure, and laboratory test results were not significantly associated with anastomotic re-leakage after stoma closure (Table 1).

### Prediction model development

**Fig. 2:**
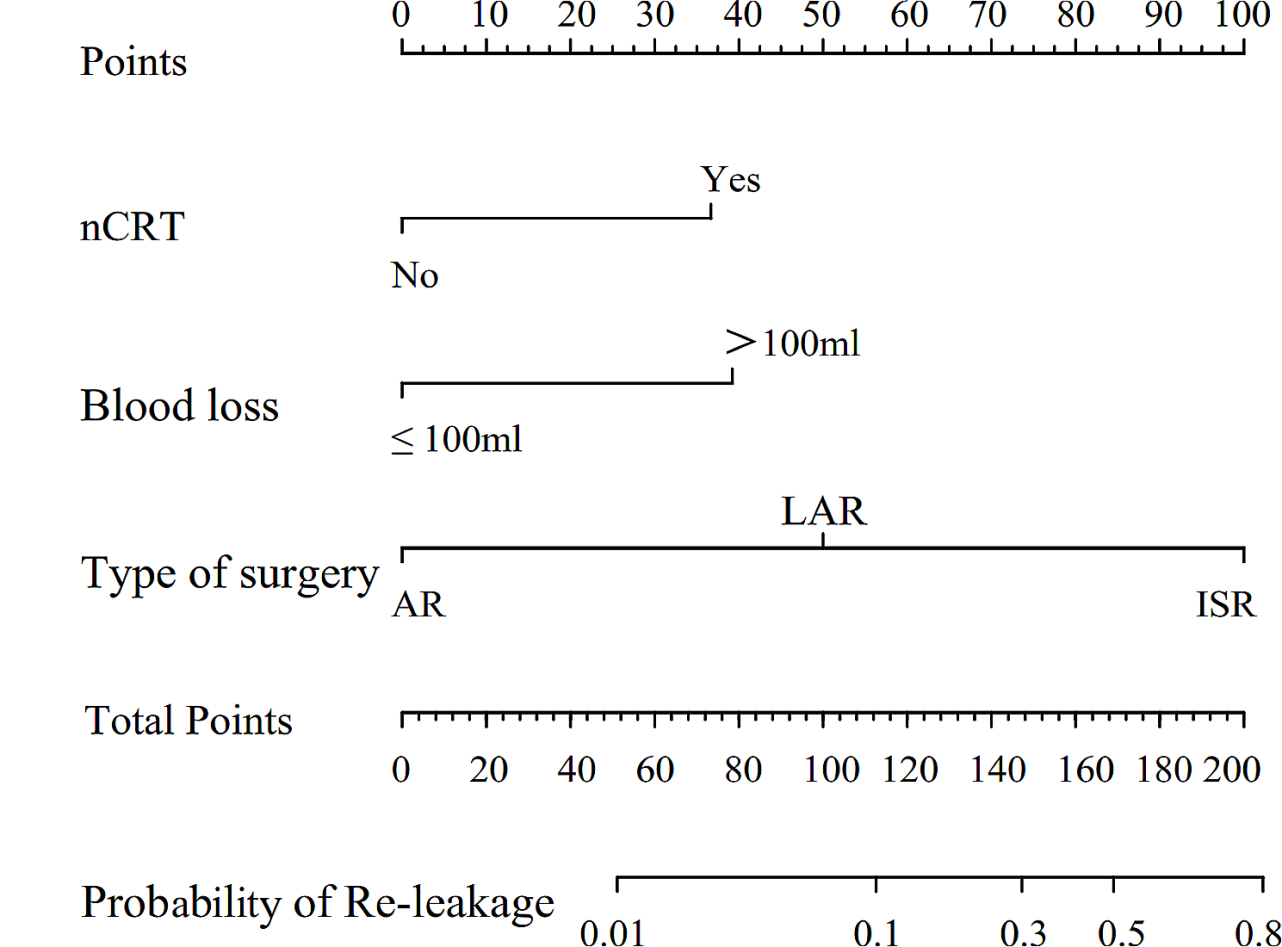
Nomogram and performance of the nomogram in our cohort The probabilities of anastomotic re-leakage following stoma closure were estimated by summing the scores for neoadjuvant chemotherapy status, blood loss, and type of surgery

Variables with a *p*-value < 0.10 in the univariate analyses included ASA score (*p* = 0.06), blood loss exceeding 50 ml (*p =* 0.02), nCRT (*p =* 0.007), and type of surgery (ISR vs. LAR, *p =* 0.001). These were selected as input variables for multivariable logistic regression. The multivariable analysis (Table 2) revealed that nCRT (OR = 4.07, 95% CI: 1.17–14.21, *p =* 0.03), blood loss exceeding 50 ml (OR = 4.52, 95% CI: 1.31–15.63, *p =* 0.02), and type of surgery (ISR vs. LAR, OR = 6.85, 95% CI: 2.01–23.36, *p =* 0.002) were independent risk factors for anastomotic re-leakage following stoma closure. Using these results, we constructed a prediction model and developed a nomogram to estimate the probability of re-leakage following stoma closure (Fig. 2). In the nomogram, blood loss exceeding 50 ml, nCRT, ISR, and LAR were assigned approximately 40, 38, 100, and 50 points, respectively. The individual scores for each risk factor were summed, and the probabilities corresponding to the total score represented probabilities of re-leakage following stoma closure.

**Table 2:** Multivariable analysis of re-leakage in patients with stoma closure

Variable	Odds ratio	95% CI	*p* value
ASA			
I–II	Reference		
III	3.25	0.66–15.96	0.15
nCRT			
No	Reference		
Yes	4.07	1.17–14.21	0.03
Blood loss			
≤ 50 ml	Reference		
>50 ml	4.52	1.31–15.63	0.02
Type of surgery			0.009
LAR	Reference		
ISR	6.85	2.01–23.36	0.002
AR	-	-	1.00

*Abbreviations*: ASA, American Society of Anesthesiologists; nCRT, neoadjuvant chemotherapy; AR, anterior resection; LAR, low anterior resection; ISR, intersphincteric resection; CI, confidence interval

### Nomogram performance

**Fig. 3:**
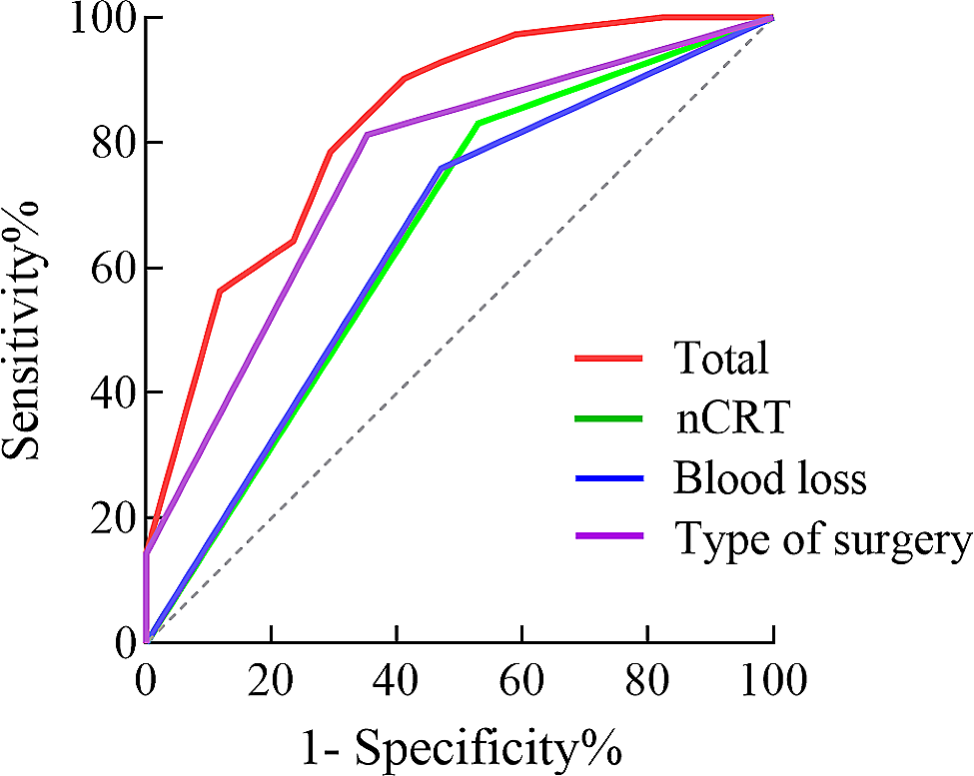
Receiver operating characteristic curve of the nomogram for the probability of anastomotic re-leakage following stoma closure in our cohort

**Fig. 4:**
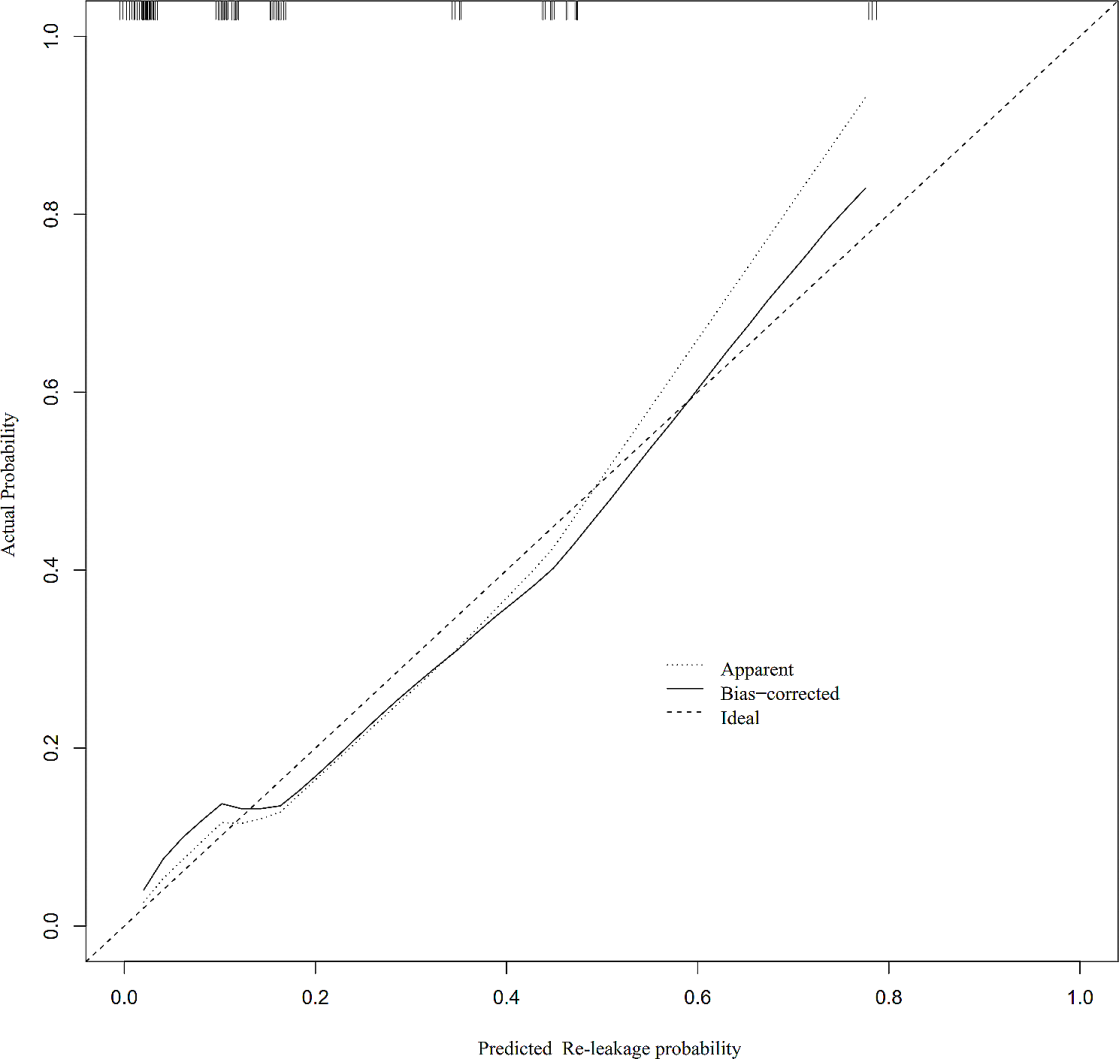
Calibration curve of the nomogram for the probability of anastomotic re-leakage following stoma closure in our cohort

The nomogram performed well in our cohort, as indicated by its predictive ability with a c-index of 0.828 in internal verification. The ROC curve showed discrimination, with an area under the ROC curve (AUC) of 0.828 (95% CI: 0.717–0.939), surpassing the predictive performance of individual risk factors in determining the probability of re-leakage after stoma closure (Fig. 3). Moreover, the calibration curve demonstrated a high level of agreement between the predicted probability of anastomotic re-leakage and the actual occurrence of re-leakage in the cohort (Fig. 4). These findings corroborate the reliability of the nomogram in accurately estimating the likelihood of re-leakage following stoma closure.

### Clinical manifestations and treatment of re-leakage following stoma closure

The median time for diagnosing re-leakage in 17 patients was 5 months (range 30 days–11 months). Clinical manifestations included presacral abscess (13 cases) and rectovaginal fistula (4 cases). All patients underwent abdominal-pelvic CT, MRI, colonoscopy, or colposcopy scans within 30–90 days after surgery to observe the condition of the anastomotic site. Radiological findings in patients with re-leakage commonly showed new gas shadows and fluid accumulation in the presacral or anastomotic area. All patients with re-leakage received active antimicrobial therapy and drainage treatment. None of the re-leakages healed successfully after 1–2 months of conservative treatment. In 14 cases, a decision was made to proceed with permanent transverse colostomy as the next step, while the other three patients lived with rectovaginal fistula or presacral abscess. Basic information and treatment measures for 17 patients with re-leakage are summarized in Table 3.

**Table 3:** Summary of 17 re-leakage cases

No.	Age	Sex	nCRT	Type of surgery	Blood loss (ml)	Time from first surgery to stoma closure (months)	Time from stoma closure to re-leakage (months)	Type of re-leakage	Investigation	Treatment for re-leakage
1	36	M	Yes	LAR	30	3	11	Presacral abscess	CT, MRI	Permanent stoma of transverse colon
2	62	F	No	LAR	50	12	6	Recto-vaginal fistula	Colonoscopy, Colposcopy	Conservative treatment
3	77	M	No	ISR	200	6	5	Presacral abscess	CT, MRI	Permanent stoma of transverse colon
4	38	M	Yes	ISR	100	13	6	Presacral abscess	CT, MRI	Permanent stoma of transverse colon
5	65	F	No	ISR	200	9	9	Presacral abscess	CT, MRI	Permanent stoma of transverse colon
6	57	M	Yes	ISR	100	6	7	Presacral abscess	CT, MRI	Permanent stoma of transverse colon
7	56	M	No	ISR	50	17	4	Presacral abscess	CT, MRI	Permanent stoma of transverse colon
8	37	F	Yes	ISR	100	16	1	Recto-vaginal fistula	Colonoscopy, Colposcopy	Conservative treatment
9	39	F	Yes	ISR	50	6	1	Recto-vaginal fistula	Colonoscopy, Colposcopy	Permanent stoma of transverse colon
10	70	M	Yes	ISR	200	8	7	Presacral abscess	CT, MRI	Permanent stoma of transverse colon
11	53	F	No	ISR	50	8	5	Presacral abscess	CT, MRI	Permanent stoma of transverse colon
12	66	M	No	LAR	200	8	6	Presacral abscess	CT, MRI	Permanent stoma of transverse colon
13	55	F	Yes	LAR	50	9	4	Recto-vaginal fistula	Colonoscopy, Colposcopy	Permanent stoma of transverse colon
14	54	M	No	ISR	200	3	3	Presacral abscess	CT, MRI	Permanent stoma of transverse colon
15	23	M	No	LAR	300	12	5	Presacral abscess	CT, MRI	Conservative treatment
16	61	M	Yes	ISR	30	12	3	Presacral abscess	CT, MRI	Permanent stoma of transverse colon
17	66	M	No	LAR	50	16	7	Presacral abscess	CT, MRI	Permanent stoma of transverse colon

*Abbreviations*: M, male; F, Female; nCRT, neoadjuvant chemotherapy; AR, anterior resection; LAR, low anterior resection; ISR, intersphincteric resection

## Discussion

This study aims to identify the risk factors in patients with rectal anastomotic re-leakage, and to develop a prediction model to estimate the probability of rectal anastomotic re-leakage after diverting stoma. Among 129 patients who met the clinical healing criteria for AL, 13.2% (17/129) developed anastomotic re-leakage following stoma closure. Only 79.4% (231/291) of patients with AL could preserve bowel continuity. This suggests that a significant proportion of patients experienced challenges in maintaining normal bowel continuity after AL. The study also identified nCRT, intraoperative blood loss during the initial surgery, and ISR as key factors associated with anastomotic re-leakage following stoma closure. Based on these predicting factors, we developed a user-friendly nomogram as a prediction model. The nomogram exhibited a c-index of 0.828, indicating its predictive ability. The nomogram in this study was only validated internally and thus lacked external validation. However, to the best of our knowledge, there is currently no existing model for predicting the development of anastomotic re-leakage after stoma closure.

One limitation of this study was its retrospective design, which may have introduced selection bias in the sample. The stoma closure rate in the overall enrolled patient population was 75%, and excluding patients who could not undergo closure due to recurrence, metastasis, or other unrelated reasons may have indirectly impacted the study results. Moreover, as a single-center study, the sample size of patients with AL and those who actually underwent stoma closure was relatively small, potentially affecting the statistical validity of the findings. Despite these limitations, this study sheds light on the relatively uncommon yet important complication of anastomotic re-leakage following stoma closure. The clinical characteristics and high-risk factors associated with this complication were thoroughly analyzed, and a prediction model was developed to estimate the probability of anastomotic re-leakage after stoma closure. Given the increasing number of patients undergoing ISR surgery after nCRT, the findings highlight the need for careful attention and professional guidance throughout the entire process of anastomotic recovery and the management of patients with AL, with the ultimate goal of preserving bowel continuity.

There is limited research on anastomotic re-leakage after stoma closure. Previous studies indicated nCRT as a risk factor for AL [16, 17]. This study’s findings suggest that nCRT continues to affect healing of the anastomosis, leading to a higher incidence of anastomotic re-leakage after stoma closure. Radiation therapy [18–20] can induce radiation enteritis in the surrounding bowel, characterized by tissue edema and local adhesions. Additionally, radiation can impact micro-vessels, causing arterial wall swelling, occlusion, and intestinal ischemia. These radiation-induced changes can impair the anastomosis healing process, increasing AL risk. Furthermore, clinical healing of AL, as confirmed by imaging studies, may actually represent a pseudo-healing of a process characterized by the formation of fibrous tissue rather than the restoration of normal mucosal intestinal wall tissue. This lack of tissue compliance can contribute to an increased risk of anastomotic re-leakage, for months or even years [21, 22].

Kitaguchi et al. [13] found that the incidence of anastomotic re-leakage following stoma closure was 25% for ISR surgery, while traditional TME surgery had a significantly lower incidence of only 5%. This study’s results support the idea that ISR is an independent risk factor for anastomotic re-leakage. This can be attributed to lower anastomosis in ISR surgery being more susceptible to compression from the anal sphincter, leading to compromised blood supply in the anastomotic area. Other studies have demonstrated that the distal rectum has fewer arterial branches [23, 24], and the surgical technique of ISR inevitably disrupts the blood supply to the distal rectum, resulting in chronic ischemia in that region. This chronic ischemia reduces the potential for successful healing of the anastomosis. Additionally, the upper levator ani hiatus provides a relatively spacious and less tissue-surrounded space, making it challenging to locally wrap and drain anastomotic leakages. This can contribute to the development of chronic pelvic abscesses or fistulas that may persist over an extended period. Furthermore, as the leakage occurs at the level of the levator ani, it is not easily detectable through enteroscopy or water-soluble contrast agent imaging before stoma closure. Once stoma closure surgery is performed, the infection or sinus will gradually worsen, leading to the recurrence of presacral pneumatocele, hydrops, and abscesses, formation of permanent anal or rectovaginal fistulas penetrating the perineum.

Even after a longer waiting period, some patients still experienced re-leakage after stoma closure. Therefore, for patients with high-risk factors, we need to be more cautious in assessing the integrity of the anastomosis before stoma closure. For some patients, a stoma might be the best choice. Of course, the pull-through colo-anal anastomosis surgical method can also be adopted, but this requires extremely high surgical skills. Indeed, if the initial anastomotic leakage could be more effectively treated, the complication of subsequent anastomotic re-leakage would no longer be a concern. In a study by Talboom and colleagues, 53 patients with anastomotic leakage following rectal cancer surgery were treated using traditional methods, while 23 cases were managed using the EVASC method. Their analysis revealed that initiating EVASC within a week post-initial surgery resulted in a 100% functional anastomosis rate. Furthermore, this approach proved more effective than traditional methods in addressing anastomotic leakage [25].

## Conclusion

Although this prediction model is helpful in guiding clinical decisions. Considering the limited effectiveness of treating anastomotic re-leakage, emphasis should be placed on its prevention. Patients with high-risk factors for re-leakage, such as nCRT, intraoperative bleeding exceeding 50 ml, or ISR surgery, require careful consideration regarding the timing of stoma closure.

## Data Availability

The database is available if properly requested and can be directly addressed to the corresponding author’s email address.

## Abbreviations

AL: Anastomotic Leakage
DS: Diverting Stoma
nCRT: Neoadjuvant Chemoradiotherapy
ISR: Intersphincteric Resection
AR: Anterior Resection
LAR: Low Anterior Resection
BMI: Body Mass Index
LCA: Left Colic Artery
CT: Computed Tomography
MRI: Magnetic Resonance Imaging
ROC: Receiver Operating Characteristic
AUC: Area Under the Curve
ASA: American Society of Anesthesiologists
CI: Confidence Interval
OR: Odds Ratio
TME: Total Mesorectal Excision
PME: Partial Mesorectal Excision

## References

[1] Tartaglia N, Pacilli M, Pavone G, Fersini A, Lizzi V, Vovola F (2021). Functional results of surgical treatment of low-ultralow rectal cancer. Ann Ital Chir.

[2] Marquardt C, Koppes P, Weimann D, Schiedeck T (2012). Laparoscopic ultralow anterior rectal resection in APPEAR technique for deep rectal cancer. Int J Colorectal Dis.

[3] Koyama M, Murata A, Sakamoto Y, Morohashi H, Hasebe T, Saito T (2016). Risk factors for anastomotic leakage after intersphincteric resection without a protective defunctioning stoma for lower rectal cancer. Ann Surg Oncol.

[4] Kawai M, Sakamoto K, Honjo K, Okazawa Y, Takahashi R, Kawano S (2022). Benefits and risks of diverting stoma creation during rectal cancer surgery. Ann Coloproctol.

[5] Matthiessen P, Hallböök O, Rutegård J, Simert G, Sjödahl R (2007). Defunctioning stoma reduces symptomatic anastomotic leakage after low anterior resection of the rectum for cancer: a randomized multicenter trial. Ann Surg.

[6] Degiuli M, Elmore U, De Luca R, De Nardi P, Tomatis M, Biondi A (2022). Risk factors for anastomotic leakage after anterior resection for rectal cancer (RALAR study): a nationwide retrospective study of the Italian Society of Surgical Oncology Colorectal Cancer Network Collaborative Group. Colorectal Dis.

[7] Karim A, Cubas V, Zaman S, Khan S, Patel H, Waterland P (2020). Anastomotic leak and cancer-specific outcomes after curative rectal cancer surgery: a systematic review and meta-analysis. Tech Coloproctol.

[8] Jiang W, Wang H, Zheng J, Zhao Y, Xu S, Zhuo S (2022). Post-operative anastomotic leakage and collagen changes in patients with rectal cancer undergoing neoadjuvant chemotherapy vs chemoradiotherapy. Gastroenterol Rep (Oxf).

[9] Herrle F, Sandra-Petrescu F, Weiss C, Post S, Runkel N, Kienle P (2016). Quality of life and timing of stoma closure in patients with rectal cancer undergoing low anterior resection with diverting stoma: a multicenter longitudinal observational study. Dis Colon Rectum.

[10] Walma MS, Kornmann VN, Boerma D, de Roos MA, van Westreenen HL (2015). Predictors of fecal incontinence and related quality of life after a total mesorectal excision with primary anastomosis for patients with rectal cancer. Ann Coloproctol.

[11] Bausys A, Kuliavas J, Dulskas A, Kryzauskas M, Pauza K, Kilius A (2019). Early versus standard closure of temporary ileostomy in patients with rectal cancer: a randomized controlled trial. J Surg Oncol.

[12] Hain E, Maggiori L, Manceau G, Zappa M, Prost à la Denise J, Panis Y (2016). Persistent asymptomatic anastomotic leakage after laparoscopic sphincter-saving surgery for rectal Cancer: can Diverting Stoma be reversed safely at 6 months?. Dis Colon Rectum.

[13] Kitaguchi D, Nishizawa Y, Sasaki T, Tsukada Y, Ikeda K, Ito M (2019). Recurrence of rectal anastomotic leakage following stoma closure: Assessment of risk factors. Colorectal Dis.

[14] Clavien PA, Barkun J, de Oliveira ML, Vauthey JN, Dindo D, Schulick RD (2009). The Clavien-Dindo classification of surgical complications: five-year experience. Ann Surg.

[15] Lo SN, Ma J, Scolyer RA, Haydu LE, Stretch JR, Saw RPM (2020). Improved risk prediction calculator for sentinel node positivity in patients with melanoma: the melanoma institute Australia nomogram. J Clin Oncol.

[16] Feeney G, Sehgal R, Sheehan M, Hogan A, Regan M, Joyce M (2019). Neoadjuvant radiotherapy for rectal cancer management. World J Gastroenterol.

[17] Ponholzer F, Klingler CP, Gasser E, Gehwolf P, Ninkovic M, Bellotti R (2022). Long-term outcome after chronic anastomotic leakage following surgery for low rectal cancer. Int J Colorectal Dis.

[18] Kato I, Dyson G, Snyder M, Kim HR, Severson RK (2016). Differential effects of patient-related factors on the outcome of radiation therapy for rectal cancer. J Radiat Oncol.

[19] Borstlap WAA, Westerduin E, Aukema TS, Bemelman WA, Tanis PJ, Dutch Snapshot Research Group (2017). Anastomotic leakage and chronic presacral sinus formation after low anterior resection: results from a large cross-sectional study. Ann Surg.

[20] Lim SB, Yu CS, Kim CW, Yoon YS, Park IJ, Kim JC (2016). Late anastomotic leakage after low anterior resection in rectal cancer patients: clinical characteristics and predisposing factors. Colorectal Dis.

[21] Qin Q, Ma T, Deng Y, Zheng J, Zhou Z, Wang H (2016). Impact of preoperative radiotherapy on anastomotic leakage and stenosis after rectal cancer resection: Post hoc analysis of a randomized controlled trial. Dis Colon Rectum.

[22] Thornton FJ, Barbul A (1997). Healing in the gastrointestinal tract. Surg Clin North Am.

[23] Scala D, Niglio A, Pace U, Ruffolo F, Rega D, Delrio P (2016). Laparoscopic intersphincteric resection: indications and results. Updates Surg.

[24] Allison AS, Bloor C, Faux W, Arumugam P, Widdison A, Lloyd-Davies E (2010). The angiographic anatomy of the small arteries and their collaterals in colorectal resections: some insights into anastomotic perfusion. Ann Surg.

[25] Talboom K, Greijdanus NG, Brinkman N, Blok RD, Roodbeen SX (2023). Comparison of proactive and conventional treatment of anastomotic leakage in rectal cancer surgery: a multicentre retrospective cohort series. Tech Coloproctol.

